# Carotid artery constriction in autoimmune hypophysitis: three case reports and literature review

**DOI:** 10.1530/EC-25-0120

**Published:** 2025-05-28

**Authors:** Sasan Darius Adib, Daniel Kopf, Brigitte Ruh-Daikeler, Rudi Beschorner, Antje Bornemann, Florian Hennersdorf, Jürgen Honegger

**Affiliations:** ^1^ Department of Neurosurgery, University of Tübingen, Tübingen, Germany; ^2^ Department of Endocrinology and Diabetology, RKH Krankenhaus Bietigheim-Vaihingen, Bietigheim-Bissingen, Germany; ^3^ Medicover Stuttgart MVZ, Stuttgart, Germany; ^4^ Institute of Neuropathology, University of Tübingen, Tübingen, Germany; ^5^ Department of Diagnostic and Interventional Neuroradiology, University of Tübingen, Germany

**Keywords:** autoimmune hypophysitis, internal carotid artery constriction, cavernous sinus, transsphenoidal, ischaemic stroke

## Abstract

**Objective:**

An intracavernous internal carotid artery constriction or occlusion (ICAc/o) has been considered an extremely rare finding in autoimmune hypophysitis (AiHy). This study aimed to analyse predictive factors for the occurrence of ICAc/o in AiHy.

**Design:**

Retrospective analysis of three of our own cases and 16 published cases.

**Methods:**

Among 15 surgically treated patients with AiHy, we identified three cases with ICAc/o via time-of-flight magnetic resonance angiography (TOF MRA) or computed tomography angiography (CTA). In addition, 16 published cases with AiHy and ICAc/o were identified via the literature search. Clinical features, treatment, and outcomes were evaluated.

**Results:**

TOF MRA revealed complete bilateral ICA occlusion (ICAo) in case 1 and incomplete bilateral ICA constriction (ICAc) in case 2. In the third case, left-sided ICAo was confirmed by CTA. None of our three patients with AiHy complicated by ICAc/o suffered brain infarction or neurological deficits. All three cases exhibited a parasellar T2 dark sign and strong dural enhancement. With our three cases included, seven of 19 published cases (36.8%) showed complete bilateral ICAo. Among these, four presented with ischaemic stroke. Eight of 19 patients (42.1%) presented with cranial nerve palsy. While all patients presented with hypopituitarism, only five had arginine vasopressin (AVP) deficiency. Interestingly, 11 patients had a recurrent course of hypophysitis.

**Conclusion:**

ICAc/o caused by AiHy appears to be more frequent than previously reported. Special attention should be paid to the carotid arteries in AiHy because of the potentially deleterious complication of ICAc/o. Cranial nerve palsy, a parasellar T2 dark sign, strong perisellar dural enhancement, and a recurrent course of hypophysitis can be considered warning signs of the occurrence of ICAc/o.

## Introduction

Hypophysitis is an umbrella term for various entities involving inflammation of the pituitary gland. It is subclassified into autoimmune hypophysitis (AiHy), with an autoimmune background, and secondary hypophysitis, which is triggered by a wide variety of local and systemic processes (1, 2, 3).

Here, we address incomplete internal carotid artery constriction (ICAc) and complete internal carotid artery occlusion (ICAo) as rare complications of AiHy. Ikeda *et al.* (4) reported the first case of AiHy with ICAo in 1990. In a review of the literature up to June 2019, Gendreitzig *et al.* (5) identified seven cases of AiHy with ICAc/o (1, 6, 7, 8, 9, 10, 11). ICAc/o is a potentially lethal complication of hypophysitis, which warrants prompt diagnosis and treatment (9). A particularly high risk of ischaemic stroke exists in cases with ICAo. On the other hand, the early diagnosis of ICAc is paramount to prevent the progressive deterioration of cerebral blood flow and stroke.

This study on AiHy with ICAc/o is based on three of our own patients and 16 previously published cases from the literature. It aimed to identify specific features of AiHy that predispose individuals to ICAc/o and to analyse the outcomes.

## Methods

### Data collection and inclusion criteria

A total of 2,204 patients with sellar region pathologies underwent transsphenoidal surgery at our department between January 2005 and February 2023. Our surgical series included 15 patients with histopathologically confirmed AiHy. Among these patients, three patients with internal carotid artery constriction or occlusion (ICAc/o) within the cavernous sinus (CS) were identified.

Clinical symptoms, diagnostic findings, treatment, and follow-up data were retrieved from patients’ files.

The study was approved by the ethics committee of the University of Tuebingen (number: 230/2023BO2).

### Radiological assessment

Preoperative and postoperative magnetic resonance imaging (MRI) of the sellar region included coronal and sagittal T1-weighted images without and with contrast, coronal T2-weighted images, and axial T2 fluid-attenuated inversion recovery images. The internal carotid arteries (ICAs) and collaterals were assessed using time-of-flight magnetic resonance angiography (TOF MRA) or computed tomography angiography (CTA).

The abbreviation ‘ICAc’ was used if the ICA showed incomplete constriction on imaging studies, and ‘ICAo’ was used if the ICA was completely occluded. ICAc/o addressed both variants.

### Endocrinological evaluation

Preoperative, postoperative, and follow-up endocrinological assessments were provided by the referring endocrinologists. In addition, oestradiol, follicle-stimulating hormone, luteinising hormone, free thyroxine, free triiodothyronine, thyroid-stimulating hormone, cortisol, prolactin, growth hormone, and insulin-like growth factor 1 levels were determined the day before surgery and 5 days after surgery during the perioperative hospital stay in our neurosurgical department in a standardised manner.

### Transsphenoidal surgery

For transsphenoidal biopsy, all patients were placed under general anaesthesia in the supine position, with the head reclined at 10°. Transsphenoidal surgery was performed under the operating microscope. A direct prenasal approach with the septum-pushover technique was used.

### Histopathological analysis

The histopathological analysis included evaluation of CD3+ T cells, CD20+ B cells, CD138+ plasma cells, IgG4+ plasma cells, reticulin, and the pituitary gland’s tissue structure.

### Literature research

A PubMed search was performed to identify publications in English or German up to December 2024. The search terms ‘hypophysitis’ combined with ‘carotid artery’ and ‘hypophysitis’ combined with ‘CS’ were used. Full-text articles were reviewed to identify published cases of AiHy and ICAc/o. Citation searches of the reviewed articles were performed to identify additional cases.

## Results

### Case 1

In 2008, a 35-year-old female patient presented with weight loss and vomiting. She had a history of schizoaffective disorder. An endocrinological evaluation revealed panhypopituitarism, and replacement therapy with hydrocortisone and L-thyroxine was commenced. She was also taking an oral contraceptive. No evidence of arginine vasopressin (AVP) deficiency was found throughout her case history. MRI showed an intrasellar lesion with a slight suprasellar extension and clear thickening of the pituitary stalk, as well as a parasellar T2 dark sign and dural enhancement suggestive of AiHy (Table 1). The clinical, endocrinological, and radiological findings remained unchanged on regular follow-up examinations until 2015.

**Table 1:** Radiological findings in three own cases with ICAc/o.

Radiological findings	Case 1	Case 2	Case 3
Internal carotid artery (ICA)	Bilateral ICA occlusion	Bilateral ICA constriction	Left-sided ICA occlusion
Parasellar T2 dark sign	Yes	Yes	Yes
Pituitary stalk thickening	Yes	No	Yes
Pituitary stalk loss of top bottom tapering	Yes	No	Yes
Strong contrast enhancement	Yes	Yes	No
Necrosis	No	No	Yes
Loss of bright spot	Yes	Yes	Yes
Compression of optic chiasm	Yes	Yes	No
Perisellar dural enhancement	Yes	Yes	Yes
Sphenoid sinus mucosa thickening	Yes	Yes	Yes

**Figure 1:**
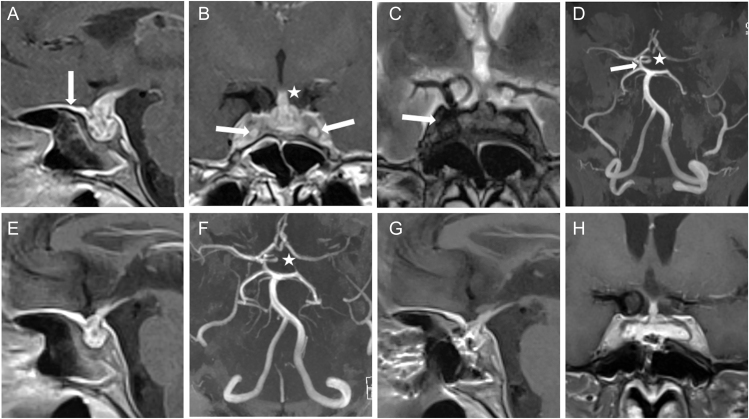
Case 1. (A) Sagittal T1-weighted MRI post-contrast at the time of bilateral ICAo diagnosis in 2015 shows intrasellar hypophysitis with suprasellar extension. Strong dural enhancement is found (arrow). (B) Coronal T1-weighted MRI post-contrast shows bilateral CS infiltration and ICA occlusion (arrows). Non-tapering of the pituitary stalk is found (asterisk). (C) T2-weighted MRI demonstrates parasellar T2 dark sign (arrow). (D) 3D TOF MRA confirms bilateral ICA occlusion (asterisk for right side) and supply of the anterior circulation mainly via a large posterior communicating artery on the left side (arrow). Following treatment with GC and short-term azathioprine, sagittal MRI (Fig. 1E) and TOF MRA (Fig. 1F) from October 2019 demonstrated size reduction of hypophysitis but persistent bilateral occlusion of the ICA. Postoperative sagittal (G) and coronal (H) T1-weighted MRI post-contrast from June 2021 demonstrate a reduced size of the pituitary lesion.

In 2015, the patient complained of blurry vision and weight gain with food cravings (Table 2). An examination of the cerebrospinal fluid (CSF) revealed slight lymphocytic pleocytosis. The serum and CSF were negative for soluble interleukin-2 receptor and angiotensin-converting enzyme. MRI revealed a significant increase in the pituitary lesion (Fig. 1). TOF MRA and carotid Doppler sonography showed bilateral ICAo (Fig. 1, Table 2). The anterior circulation was supplied by collaterals from the vertebrobasilar circulation via a large left and small right posterior communicating artery (Fig. 1). No signs of brain infarction were detected. Under the presumptive diagnosis of AiHy, treatment with methylprednisolone was started, with prompt improvement of the visual disturbance (Table 3). However, treatment had to be discontinued after 10 days because of the exacerbation of her psychosis.

**Table 2:** Clinical presentation in the 16 literature cases and three own cases with AiHy and ICAc/o.

	Authors/year	^†^Age/gender	Symptoms	^‡^Duration (months)	Optomotor nerve palsy	Hypopituitarism	AVP deficiency	ICAc/o	Infarction (MRI)	Neurological deficit
1	Ikeda *et al.* (1990) (4)	45 f	Blurred vision, headache, abnormal thirstiness	36	No	Panhypopit	‘Abnormal thirstiness’	Bilat ICAo	None	None
2	Supler *et al.* (1992) (12)	56 m	Retroorbital headache	5	Right abducens nerve palsy	Panhypopit	No	Right ICAo	None	None
3	Leung *et al.* (2004) (8)	57 m	Headache, decreased libido, lethargy	18	No	Hypothyroidism, hypogonadism	No	Bilat ICAo	Bilat ischaemic infarction	Severe impairment, prolonged rehabilitation
4	Melgar *et al.* (2006) (9)	38 f	Initial: diplopia, headache; at the time of ICAo diagnosis: headache	22	Right abducens nerve palsy	Slightly low cortisol, otherwise normal	No	Bilat ICAo	Bilat fronto-parietal ischaemic infarction	Perioral tingling, slurred speech, weakness and hypoesthesia in lower extremities
5	Nakata *et al.* (2010) (13) ‘case 1’	38 f	Headache, vomiting, polyuria, polydipsia	11	No	Hypogonadism	Yes	Left ICAc	None	None
6	Nakata *et al.* (2010) (13) ‘case 2’	53 m	Decreased libido	NA	No	Hypogonadism	No	Left ICAc	None	None
7	Nakata *et al.* (2010) (13) ‘case 4’	36 m	Decreased libido, polyuria, polydipsia	8	No	Hypogonadism	Yes	Right ICAc	None	None
8	Peruzzotti-Jametti *et al.* (2012) (10)	55 f	Headache, nausea	10	No	Not mentioned	No	Bilat ICAo	Left fronto-parietal ischaemic infarction	Slurred speech, right hemiparesis
9	Zoeller *et al.* (2012) (11)	41 m	Initial: left eye pain, visual loss; at the time of ICAc diagnosis: right eye pain, visual loss, headache, dizziness, fatigue	>6	No	Hypocortisolism (iatrogenic), hypogonadism	No	Right ICAc	None	None
10	Kanoke *et al.* (2013) (6)	53 f	Dull headache, retroorbital heavy feeling	NM	No	Hypothyroidism, hypogonadism	No	Right ICAc	None	None
11	Katsiveli *et al.* (2016) (7)	48 f	Headache, muscle weakness	‘Previous months’	No	Panhypopit	No	Bilat ICAo	Small fronto-parietal ischaemic infarction	Slurred speech, right-sided hemiparesis
12	Pekic *et al.* (2018) (1)	42 f	Headache, diplopia, amenorrhoea, dizziness	24	Right abducens nerve palsy	Hypocortisolism	No	Subtotal right ICAc, left ICAo	NM	None
13	*Lin *et al.* (2020) (14)	50 f	Initial: headache, diplopia; at the time of ICAo diagnosis: again headache, diplopia	24	Minor bilateral abducens nerve palsy	Hypogonadism	No	Bilat ICAo	None	None
14	Gendreitzig *et al.* (2020) (5)	29 f	Headache, nausea, vomiting, fatigue, adrenal crisis	137	‘Diplopia’	Panhypopit	Yes	Bilat ICAc	None	None
15	Rojas *et al.* (2022) (16)	54 f	Intense headache	52	No	Hypothyroidism hypogonadism	Yes	Left ICAo	None	None
16	Watanabe *et al.* (2023) (15)	75 f	Initial: nausea, vomiting; at the time of ICAc diagnosis: right ocular pain	10	Right oculomotor nerve palsy	Panhypopit	Yes	Severe right ICAc	NM	None
17	Own case 1	35 f	Initial: weight loss, vomiting; at the time of ICAo diagnosis: blurred vision, weight gain	84	Left abducens nerve palsy	Panhypopit	No	Bilat ICAo	None	None
18	Own case 2	48 f	Headache, double vision, nausea, adynamia	0.5	Right oculomotor nerve palsy & abducens nerve palsy	Hypothyroidism hypocortisolism	No	Bilateral ICAc	None	None
19	Own case 3	31 f	Tiredness, muscle pain, amenorrhoea, headache, weight loss	18	No	Panhypopit	No	Left ICAo	None	None

* Previously reported by Waldie *et al.* (2019) (17).

^†^ Age at initial symptom onset.

^‡^ Duration from initial symptom onset to diagnosis of ICAc/o.

Abbreviations: Bilat, bilateral; ICAo, internal carotid artery occlusion; ICAc, internal carotid artery constriction; NM, not mentioned; Panhypopit, panhypopituitarism.

**Table 3:** Treatment in the 16 literature cases and three own cases with AiHy and ICAc/o.

	Authors/year	Age/gender	Transsphenoidal surgery	Histopathological diagnosis	Recurrent course of hypophysitis	Treatment for hypophysitis and ICAc/o	Specific treatment for ICAc/o
1	Ikeda *et al.* (1990) (4)	45 f	Partial removal	Lymphocytic hypophysitis	No	NM (no follow-up)	None
2	Supler *et al.* (1992) (12)	56 m	Biopsy	Lymphocytic hypophysitis	No	None	None
3	Leung *et al.* (2004) (8)	57 m	‘Surgery’	Granulomatous hypophysitis	No	NM	None
4	Melgar *et al.* (2006) (9)	38 f	Partial resection	Lymphocytic hypophysitis	Yes	Prednisone pulse (twice)	Bilateral superficial temporal artery-distal middle cerebral artery bypass surgery
5	Nakata *et al.* (2010) (13) ‘case 1’	38 f	Biopsy	Lymphocytic hypophysitis	NA	NA	NA
6	Nakata *et al.* (2010) (13) ‘case 2’	53 m	Biopsy	Lymphocytic hypophysitis	No	None	None
7	Nakata *et al.* (2010) (13) ‘case 4’	36 m	No surgery	None (clinical diagnosis of lymphocytic hypophysitis)	NA	NA	NA
8	Peruzzotti-Jametti *et al.* (2012) (10)	55 f	Biopsy	Lymphocytic hypophysitis	Yes	‘Steroid therapy’	None
9	Zoeller *et al.* (2012) (11)	41 m	Biopsy	Lymphocytic hypophysitis	Yes	‘High dose steroids’ (twice), prednisone pulse (twice), ‘another course of steroids’	Acetylsalicylic acid
10	Kanoke *et al.* (2013) (6)	53 f	Biopsy	IgG4-related hypophysitis	No	High-dose hydrocortisone pulse	None
11	Katsiveli *et al.* (2016) (7)	48 f	No surgery	None (clinical diagnosis of lymphocytic hypophysitis)	Yes	Methylprednisolone, methylprednisolone escalated, azathioprine	Anticoagulant treatment
12	Pekic *et al.* (2018) (1)	42 f	Biopsy & decompression	Lymphocytic hypophysitis	Yes	Prednisone pulse, methylprednisolone (switched to prednisone), azathioprine, gamma knife radiosurgery	None
13	*Lin *et al.* (2020) (14)	50 f	Biopsy	Lymphocyctic hypophysitis	Yes	Methylprednisolone (3 pulses), azathioprine, rituximab (twice), mycophenolate mofetil	None
14	Gendreitzig *et al.* (2020) (5)	29 f	Resection (twice)	Granulomatous hypophysitis	Yes	Prednisolone pulse, azathioprine, rituximab	Acetylsalicylic acid
15	Rojas *et al.* (2022) (16)	54 f	Biopsy	IgG4-related hypophysitis	Yes	Methylprednisolone (twice), cyclophosphamide, rituximab	None
16	Watanabe *et al.* (2023) (15)	75 f	Biopsy	Lymphocytic hypophysitis	Yes	Methylprednisolone (three times), azathioprine	Acetylsalicylic acid
17	Own case 1	35 f	Biopsy	Lymphocytic hypophysitis	Yes	Methylprednisolone (three times), azathioprine	None
18	Own case 2	48 f	Biopsy	Lymphocytic hypophysitis	Yes	Prednisolone	None
19	Own case 3	31 f	Biopsy	Lymphocytic hypophysitis	No	Prednisolone pulse	None

* Previously reported by Waldie *et al.* (2019) (17).

In October 2019, the patient experienced another episode, with a severe decline in vision in her left eye and a headache that resolved with 500 mg methylprednisolone over 3 days, which precipitated another psychotic episode. She also received short-term treatment with azathioprine, which had to be terminated because of deteriorated haematopoiesis (Table 3). Following medical treatment, MRI demonstrated reduction of lesion size, and TOF MRA showed persistence of bilateral ICA occlusion (Fig. 1).

**Figure 2:**
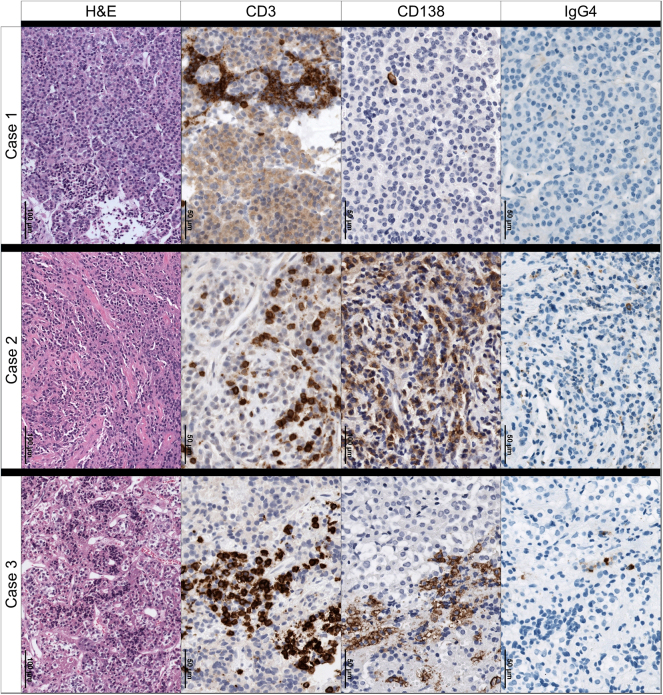
In all three cases, the pituitary gland showed a lymphocytic infiltration with CD3+ T-lymphocytes and variable numbers of CD138+ plasma cells. IgG4+ plasma cells were absent or only rarely found, thus not indicating IgG4-related hypophysitis.

In October 2020, the patient presented to our neurosurgical department due to newly developed double vision. Left-sided incomplete abducens nerve palsy was found. MRI showed a slight increase in the pituitary lesion. Because of the severe and recurrent course of her condition, we decided to perform a biopsy for a definite diagnosis (Table 3). During transsphenoidal surgery, greyish and firm intrasellar tissue and atrophic anterior pituitary tissue were found. Histopathological evaluation of the biopsy showed infiltration of the anterior pituitary gland with CD3+ T-lymphocytes and, to a lesser extent, with CD20+ B-lymphocytes, confirming the diagnosis of LyHy. There were only single CD138+ plasma cells and no IgG4+ plasma cells (Fig. 2, Table 3).

In the postoperative course, the abducens nerve palsy spontaneously regressed (Table 4). Postoperative MRI showed a clear decrease of the space-occupying lesion (Fig. 1). In November 2024, she suffered a recurrence with visual decline, which regressed after 4 days of high-dose prednisolone treatment. Currently, she declines non-steroidal immunosuppressive treatment for fear of side effects.

**Table 4:** Final outcome in the 16 literature cases and three own cases with AiHy and ICAc/o.

	Authors/year	Age/gender	ICAc/o	Neurological deficit	Optomotor nerve palsy	Hypopituitarism	Pituitary lesion size	General outcome	^†^Follow-up
1	Ikeda *et al.* (1990) (4)	45 f	NM (no follow-up)	NA	NA	NA	NA	NA	0 months
2	Supler *et al.* (1992) (12)	56 m	Unchanged	None	Completely resolved	Panhypopit	Unchanged	Headache not mentioned	6 months
3	Leung *et al.* (2004) (8)	57 m	NM	Relative independence, unable to return to work	NM	New hypocortisolism	NM	No headache	107 months
4	Melgar *et al.* (2006) (9)	38 f	Patent right-sided anastomosis, good cerebrovascular reserve	Symptoms resolved	Completely resolved	Unchanged	Unchanged mass	Returned to her previous occupation	16 months
5	Nakata *et al.* (2010) (13) ‘case 1’	38 f	NA	NM	NM	NM	NM	NM	11 months
6	Nakata *et al.* (2010) (13) ‘case 2’	53 m	Unchanged	NM	NM	NM	NM	NM	8 months
7	Nakata *et al.* (2010) (13) ‘case 4’	36 m	NA	NA	NA	NA	NA	NA	18 months
8	Peruzzotti-Jametti *et al.* (2012) (10)	55 f	Unchanged	NM	None	NM	NM	NM	NM
9	Zoeller *et al.* (2012) (11)	41 m	Not mentioned	None	None	Hypocortisolism, hypogonadism	Regression	Vision improved, constitutional symptoms significantly improved	3 months
10	Kanoke *et al. *(2013) (6)	53 f	Regressed	None	None	NM (‘taking 0.5 mg dexamethasone’)	NM	Good condition, symptom free	6 months
11	Katsiveli *et al.* (2016) (7)	48 f	Unchanged	Probably none (but not fully clear)	None	Restored	Reduction of pituitary mass	No clinical evidence of disease	12 months
12	Pekic *et al.* (2018) (1)	42 f	Not mentioned	None	None	Panhypopit	Regressed	Symptom free	48 months
13	*Lin *et al.* (2020) (14)	50 f	Unchanged	None	None	Hypogonadism	Unchanged	Remains well, headaches improved, no eye problems	30 months
14	Gendreitzig *et al.* (2020) (5)	29 f	Regressed	None	None	Unchanged	Unchanged	Symptom free, working fulltime	42 months
15	Rojas *et al.* (2022) (16)	54 f	Unchanged	None	None	NM	Still pituitary mass	Asymptomatic	29 months
16	Watanabe *et al.* (2023) (15)	75 f	Persistent	None	Improved	NM	Decreased	NM	Duration NM
17	Own case 1	35 f	Persistent	None	Regressed	Unchanged	Decreased	Clinically stable	115 months
18	Own case 2	48 f	Regressed	None	Regressed	Hypocortisolism recovered	Minimal regression	Feeling well	55 months
19	Own case 3	31 f	Persistent	None	None	Unchanged	Regressed	Feeling better	23 months

* Previously reported by Waldie *et al.* (2019) (17).

^†^ After diagnosis of ICAc/o.

Abbreviations: NA, not available; NM, not mentioned; Panhypopit, panhypopituitarism.

### Case 2

**Figure 3:**
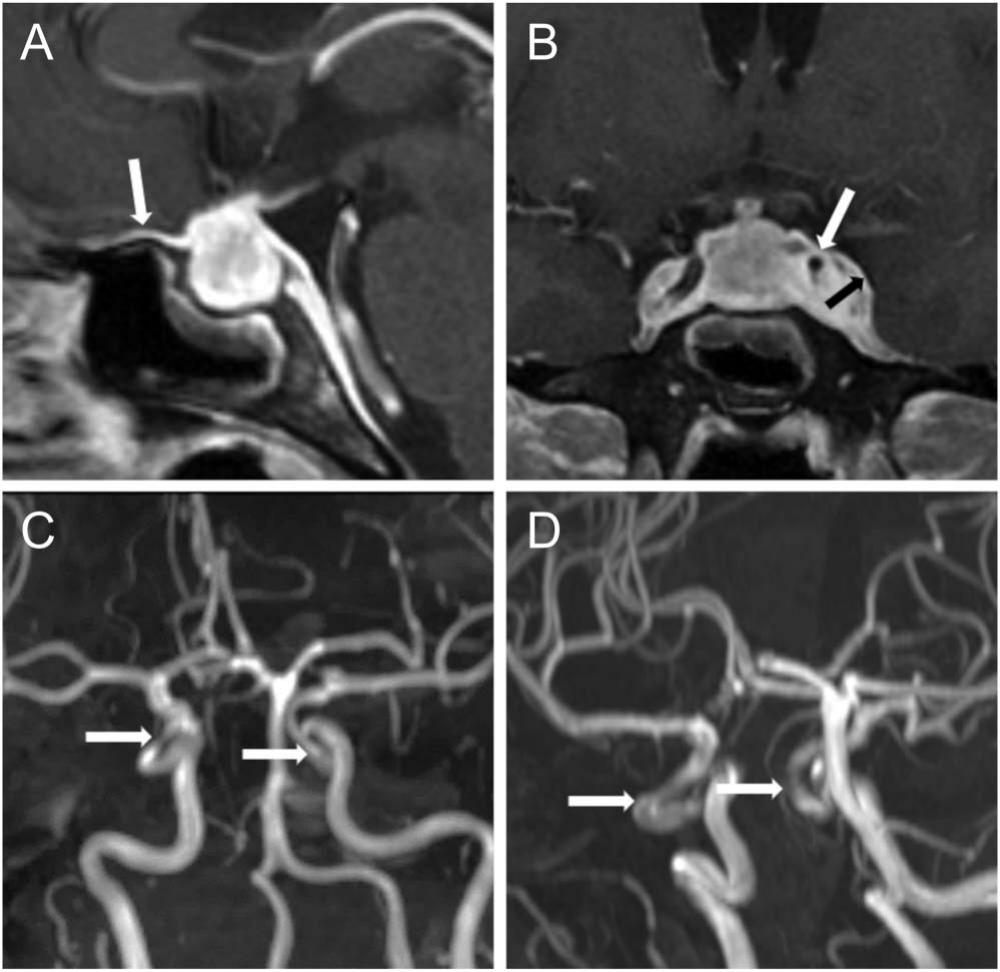
Case 2. (A) Sagittal T1-weighted MRI post-contrast at initial presentation shows intrasellar hypophysitis with suprasellar extension. Strong dural enhancement (arrow) and swelling of the sphenoid sinus mucosa are found. (B) Coronal T1-weighted MRI post-contrast shows bilateral CS infiltration with bulging of the CS towards the temporal lobe (black arrow) and narrowing of the left ICA (white arrow). (C) 3D TOF MRA confirms bilateral constriction of the ICA within the CS (arrows). (D) Follow-up 3D TOF MRA shows regression of ICA constriction with normal ICA diameters.

In May 2020, a 48-year-old female patient presented to our neurosurgical department with a 2-week history of severe headache and neck pain followed by double vision and slight nausea a few days later (Table 2). She also reported increasing adynamia. Her menstrual cycle was normal. An endocrinological evaluation showed mild secondary hypothyroidism. Prolactin was mildly elevated. There was no evidence of adrenal insufficiency, growth hormone deficiency, or hypogonadism. Surprisingly, serum cortisol became subnormal 3 days later, and replacement therapy with hydrocortisone was commenced (Table 2). An ophthalmological examination revealed a right-sided incomplete oculomotor nerve palsy and abducens nerve palsy (Table 2). The visual fields were normal. MRI showed a symmetrical intrasellar and suprasellar lesion with homogeneous contrast uptake and a slight elevation of the optic chiasm (Fig. 3). A parasellar T2 dark sign and broadening of the CS were found. MRI and TOF MRA revealed bilateral intracavernous narrowing of the internal carotid artery that was more pronounced on the left side (Fig. 3). Lumbar puncture revealed a count of 12 lymphocytes/μL and was otherwise normal. The suspected diagnosis of LyHy was confirmed by transsphenoidal surgery, which was performed in June 2020 (Fig. 2, Table 3). Immunoglobulin G4 (IgG4)-positive plasma cells were rarely found, excluding a diagnosis of IgG4-related hypophysitis. Postoperatively, the ocular nerve palsies rapidly regressed (Table 4). Therefore, immunosuppressive treatment was withheld. On postoperative TOF MRA, complete regression of ICA narrowing was found (Fig. 3). FDG-PET/MRI showed no evidence of systemic IgG4-related disease. Two months after surgery, a short Synacthen test showed a normalised adrenal axis, and hydrocortisone was discontinued. In August 2024, the patient suffered recurrence of double vision that regressed after treatment with 1,000 mg prednisolone over 5 days. At present, she is feeling well and has a normal ophthalmological status (Table 4).

### Case 3

**Figure 4:**
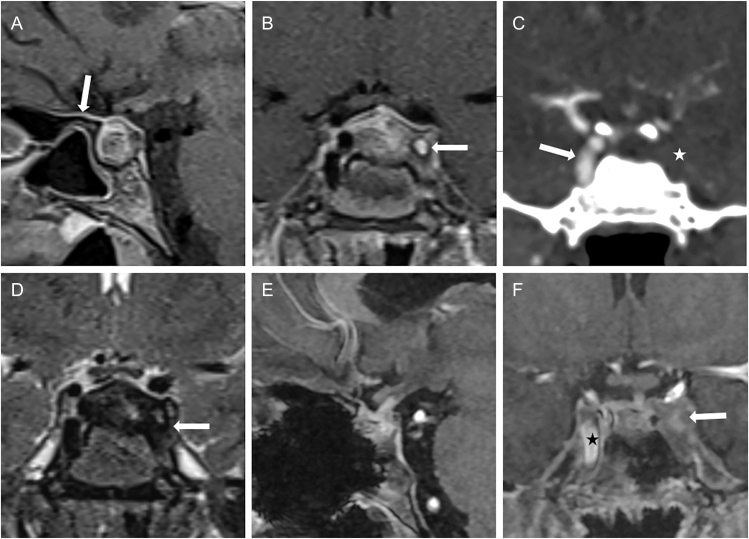
Case 3. (A) Sagittal T1-weighted MRI post-contrast at initial presentation shows an intrasellar space-occupying lesion. Strong dural enhancement is found (arrow). (B) Coronal T1-weighted MRI post-contrast shows occlusion of the left ICA (white arrow). (C) CT angiography confirms complete occlusion of the left ICA in the cavernous segment (asterisk) and a perfused right ICA with normal calibre (arrow). (D) T2-weighted MRI demonstrates parasellar T2 dark sign (arrow). (E) Sagittal T1-weighted MRI post-contrast shows reduced size of the lesion following surgery and GC therapy. (F) Coronal T1-weighted MRI post-contrast shows persistent occlusion of the left internal carotid artery following surgery and GC therapy (white arrow) and a regular flow signal in the normal-sized right internal carotid artery (black asterisk).

A 31-year-old female patient presented to an external endocrinological department in January 2023 with tiredness, muscle pain, secondary amenorrhoea, and headache (Table 2). She reported long-standing arterial hypertension and had lost 7 kg of body weight in the past 18 months. An endocrinological evaluation revealed panhypopituitarism, with a serum cortisol level as low as 0.8 μg/dL (Table 2). Fluid intake and output were within normal limits. The patient was started on 30 mg hydrocortisone and 50 μg L-thyroxine per day. Her neurological status was normal. MRI showed an intrasellar and slightly suprasellar lesion with a central cystic or necrotic appearance, enlargement of the pituitary stalk, and a parasellar T2 dark sign (Fig. 4). A presumptive diagnosis of LyHy was made. MRI demonstrated complete occlusion of the left ICA within the CS, which was confirmed by CTA (Fig. 4). No evidence of cerebral ischaemia was found on MRI. Because of the serious finding of ICAo, we recommended a transsphenoidal biopsy to clearly confirm the diagnosis. Thus, the patient was referred to our neurosurgical department and a biopsy was performed via a transsphenoidal approach in January 2023 (Table 3). The histopathological examination confirmed the diagnosis of LyHy with both B-lymphocytes and T-lymphocytes, with the latter prevailing. Focally, several CD138+ plasma cells were noted, among them only singular IgG4+ plasma cells (Fig. 2, Table 3).

Having confirmed the diagnosis, prednisolone pulse therapy was commenced in February 2023 with a starting dose of 50 mg daily (Table 3). In November 2023, Doppler sonography and CTA showed persistent ICAo and normal perfusion of the right ICA, as well as normal cerebral perfusion. Under prednisolone treatment, the patient’s general well-being was much improved. As rapid tapering of prednisolone was not tolerated, the dose was slowly reduced and reached a replacement dose in October 2024. Postoperative endocrinological follow-up examinations showed unchanged panhypopituitarism. MRI from December 2024 showed regression of the pituitary lesion, but the persistence of left-sided ICAo (Table 4; Fig. 4).

## Review of the literature

### Clinical presentation

In the literature, 16 cases of AiHy with ICAc or ICAo were reported between 1990 and 2024 (1, 4, 5, 6, 7, 8, 9, 10, 11, 12, 13, 14, 15, 16, 17). The present study adds three cases (Tables 2, 3, 4). The average age at initial presentation with AiHy was 46 years, and the average age at the time of ICAc/o diagnosis was 48 years. Interestingly, an association with pregnancy was not found in any of the cases.

Of 19 patients, eight patients presented with cranial nerve (CN) palsy as a clinical sign of CS involvement (Table 2). The CNs III and VI were affected, with a predominance of CN IV (abducens nerve). Four patients (cases 1, 2, 9, 17) presented with chiasmal syndrome.

All patients suffered from some degree of hypopituitarism, with seven suffering from panhypopituitarism. Interestingly, only five patients suffered clear AVP deficiency (Table 2).

Bilateral ICAo was observed in seven cases (36.8%); see Table 2. Four of them suffered ischaemic stroke with neurological deficits (Table 2). Of note, three patients with bilateral ICAo remained free of cerebral infarction and were neurologically intact. None of the patients with incomplete ICAc or unilateral pathology suffered infarction or neurological deficits.

Eleven of 19 patients (57.9%) had a recurrent course of hypophysitis (Table 3). In eight patients, ICAc/o was detected during a recurrent episode of hypophysitis.

### Treatment

The subtype of hypophysitis was confirmed in 17 cases by transsphenoidal biopsy. Biopsy was undertaken in 12 patients at the time when ICAc/o was detected, in three patients at an earlier stage (cases 4, 14, 16), and in one patient at a later stage (case 17). The time of surgery was not available for one patient. Among the 17 cases with biopsy, 13 cases suffered from LyHy, two had GrHy, and two had IgG4-related hypophysitis (Table 3). In our own cases, the histopathological findings were not different from typical findings in AiLy without ICAc/o. In particular, no evidence of severe destructive inflammation indicating aggressiveness was found (Fig. 2).

Thirteen of 15 patients with sufficient treatment information were treated with glucocorticoids (GC), some with several GC pulses (Table 3). From 2016 onward, seven patients were treated with non-steroidal immunosuppressive medication (Table 3). In one patient with a difficult clinical course, stereotactic radiosurgery with Gamma Knife was successfully performed.

In six cases, clear evidence exists that ICAc/o influenced the management (bypass surgery in one case and more aggressive treatment with GC and/or non-steroidal immunosuppression in five cases). In five additional cases, ICAc/o has likely influenced the management with more extensive immunosuppressive treatment. In two of these cases, incomplete ICAc was reversible under intensified treatment.

In one patient, reduced cerebral perfusion was treated with a bilateral cerebral bypass surgery (Table 3). Anticoagulant or antiplatelet treatment was reported in four patients.

### Outcome

The mean follow-up period after diagnosis of ICAc/o was 32.2 months (range 0–115 months; median 12 months).

ICAo was irreversible in all affected patients. In contrast, incomplete ICAc regressed in three patients (Table 4). One out of four patients with cerebral stroke had an adverse neurological outcome. The outcome of CN palsies was favourable (Table 4). A decrease in the pituitary lesion during follow-up was reported in seven patients, unchanged size in five patients, and no information on final lesion size in seven patients. The overall outcome was reported to be favourable in 12 patients and not reported in six patients, while one patient remained disabled following an ischaemic stroke.

Posterior pituitary function remained unchanged during follow-up in all cases (Table 4). Anterior pituitary function was restored in only one patient (case 11).

## Discussion

ICA occlusion or constriction is a serious complication of AiHy. It can cause disabling neurological deficits or even result in fatal outcomes. We report three of our cases of AiHy with ICAc/o and 16 previous cases identified by the literature search. Our study aimed to characterise cases of ICAc/o and to identify predictive factors and warning signs that allow early diagnosis.

In pathologies with CS involvement, the serious sequelae of arterial constriction and occlusion may occur (4, 18, 19, 20). ICAc/o is mainly found in benign neoplasms, AiHy, and fungal or bacterial infections (5, 21, 22, 23).

In an MRI study, Molitch *et al.* (24) analysed ICA compression in 83 patients with pathologies encasing the ICA. Only 1/58 (1.7%) pituitary adenomas but 7/25 (28%) of non-pituitary adenoma lesions showed ICA compression.

Katsiveli *et al.* (7) pointed out that, as of 2016, only two cases of bilateral ICAo along with aggressive forms of LyHy had been reported. They concluded that bilateral ICAo associated with AiHy is an exceptionally rare clinical entity. The number of cases has rapidly increased since then, with 19 cases reported here. Gendreizig *et al.* (5) analysed the entities causing ICAc/o in 36 published cases and found that 19% of ICAc/o was caused by hypophysitis, making it the second most common cause after pituitary adenomas. They concluded that hypophysitis is not as rare a cause of ICAc/o as previously considered. It was likely underreported previously because of less sophisticated detection techniques.

Given a reported yearly incidence of AiHy of only one person per 9 million (25), the relative prevalence of ICAc/o among patients with AiHy is much higher than in pituitary adenomas.

ICAc/o is a potentially life-threatening sequela of CS invasion and is associated with a high rate of neurological deficits secondary to cerebral stroke (5, 9). It is of note that none of the reported patients with ICAo secondary to hypophysitis had a fatal outcome, but fatalities have been described in other pathologies causing ICAo (5, 21). Therefore, ICAo must be regarded as a potentially life-threatening complication of hypophysitis. Early diagnosis is paramount and can be life-saving. Our review of the literature showed that four of seven patients with AiHy and bilateral ICAo suffered a cerebral stroke, while three patients had neither radiological nor clinical evidence of cerebral infarction. We and others postulate that ICAc can slowly progress in hypophysitis, allowing development of arterial collaterals, and is therefore compensated without leading to cerebral infarction in some cases (4, 5, 7, 8, 9). This theory is supported by the finding that most reported cases had a prolonged course of hypophysitis, and ICAo was not found at the initial diagnosis but at the stage of recurrence. In the four patients with symptomatic ICAo, neurological signs suggestive of stroke also developed with delay. The time span from initial symptoms of hypophysitis to development of neurological symptoms was 18, 22, 10 months, and greater than 9 months, respectively. This finding supports the hypothesis that chronic or recurrent inflammation rather than acute inflammation caused ICAc/o.

The pathophysiology of ICAc/o is not fully understood. It obviously occurs because of spread of the inflammatory process to the CS that hosts the carotid artery (7). However, it is unclear whether ICAc/o is purely caused by compression of the ICA. Infiltration and consecutive thickening of the ICA wall is another factor that likely contributes to ICAc/o (6, 16).

CS infiltration by AiHy may cause diplopia, eye pain, ptosis, facial numbness, or trigeminal neuralgia. A considerable number of hypophysitis patients with ICAc/o suffered from optomotor deficits (Table 2). This is a plausible finding, as the CS hosts CNs III, IV, and VI, which regulate eye movement. CN VI (abducens nerve), with its course through the CS, was predominantly affected. Given these findings, optomotor nerve palsy in AiHy should alert clinicians to the need for a close look at the CS and the ICA. In contrast to the cases with ICAc/o, optomotor nerve palsies were found in fewer than 10% of the patients with AiHy without ICAc/o (26). If the ICA is conspicuous on MRI, further examination – such as via TOF MRA, CTA, and digital subtraction angiography – is indicated. TOF MRA is our preferred imaging technique if ICAc/o is suspected. It should also be performed in the follow-up of patients with confirmed ICAc.

Another conspicuous finding of hypophysitis with ICAc/o is that only one in four of the patients suffered from obvious AVP deficiency, which contrasts with the value of 47% in a recent meta-analysis of all published cases with LyHy (27). This observation suggests that the cases with ICA narrowing mainly represent the lymphocytic adenohypophysitis variant.

AiHy typically presents as a symmetrical pituitary mass with homogeneous and strong contrast enhancement (3, 28). The most characteristic radiological sign of hypophysitis is the thickening of the stalk, reported in up to 86% of the cases (26, 28). Only a minority of 10% exhibit CS involvement (27).

Nakata *et al.* (13) described the parasellar T2 dark sign as a typical finding in AiHy with CS involvement and found a 100% specificity in differentiating AiHy from pituitary adenomas. The T2 dark sign is found around the CS and pituitary gland and within the CS. Most likely, it reflects fibrotic changes and thickening of dural structures caused by the inflammatory process of hypophysitis (13). We also found a parasellar T2 dark sign in all three of our cases. Therefore, it is important to pay attention to the parasellar T2 dark sign in every case with AiHy to identify CS infiltration with the potential risk of ICAc/o. The T2 dark sign was also shown on the MRI of some published cases. Of note, a pronounced perisellar dural enhancement (‘dural tail’) was found in our three cases and other published cases with ICAc/o (6, 7). It can be considered a further sign of spread outside the pituitary fossa boundaries and should be looked for in AiHy (6, 28).

High-dose glucocorticoid (HD-GC) administration is considered the mainstay of AiHy treatment and is associated with a high initial response rate (2, 29). However, it is associated with a higher recurrence rate when compared to observation (27, 29). Non-steroidal immunosuppressive drugs, such as azathioprine or rituximab, are also proven to be effective and are usually used if treatment with GCs fails (1, 5, 16).

In a German cohort study on AiHy without ICAc/o, 30% of patients were initially treated with HC-GCs and 1% with non-steroidal immunosuppressive medication (29). Forty percent did not require such treatment and were solely kept under observation. Similar results were found in an observational study of 113 patients with AiHy, with initial observation in 52.2% of patients and HC-GC therapy in 18.6% (30).

In contrast, among the reported patients with ICAc/o, the need for immunosuppressive medication was much higher, with HD-GCs in 92.9% and non-steroidal immunosuppressive medication in 38.8%. This underlines that hypophysitis complicated by ICAo/n is mostly found in aggressive and recurrent clinical forms of hypophysitis, necessitating multiple medical treatments.

Based on the findings in our review, we recommend resolute treatment of hypophysitis in the presence of the identified predictors for ICAo/c (i.e. CN palsy, parasellar T2 dark sign, strong perisellar dural enhancement, and recurrent course of hypophysitis) not only for patients who already present with incomplete ICAc but also for patients without radiological signs of ICA abnormalities. Similarly, Peruzzotti-Jametti *et al.* (10) have pointed out, ‘In our case, in fact, early testing could have prevented the bilateral occlusion of the ICAs, stroke, and subsequent disability’. However, the evidence from our literature review suggests that resolute treatment (with GC and/or non-steroidal immunosuppression) is not absolutely necessary if bilateral ICAo has already occurred.

In all but two cases, the subtype of hypophysitis was confirmed by transsphenoidal biopsy (Table 3). In contrast, surgery has been used in only 25–30% of the cases of AiHy without ICAc/o (29, 30). We hypothesise that the aggressive clinical course of hypophysitis and/or the impending threat because of ICAc/o were the reasons to confirm the diagnosis by biopsy in the reported cases. The threat of ICAc/o was also an indication for surgery in our three patients.

In cases with hypophysitis and ICAc/o, treatment to improve cerebrovascular perfusion is an additional important issue. In the case of insufficient collaterals with symptoms due to hypoperfusion, bypass surgery should be discussed. In the literature, reperfusion of the ICA never occurred once complete occlusion of the ICA was detected. In contrast, incomplete ICAc resolved in three of nine cases. One of the three patients with regression of ICAc had received high-dose hydrocortisone pulse therapy (6), one was treated with prednisolone, azathioprine, and rituximab (5), and in our case 2, ICA narrowing resolved spontaneously.

Katsilveli *et al.* (7) concluded that prompt identification of artery occlusion in hypophysitis is critical and should lead to the optimisation of medical management, thereby preventing clinical deterioration with stroke or other grave consequences.

The literature shows that hypophysitis with ICAc/o is a threatening disorder that frequently requires more than the standard treatment for hypophysitis. We strongly recommend a biopsy of the lesion, which is easily performed via a transsphenoidal approach, if there is any doubt about the precise diagnosis. This will allow the targeted treatment of the underlying disorder. An impending ICAo necessitates active treatment instead of watchful waiting.

## Conclusion

ICAc/o in AiHy is apparently more frequent than previously reported. We add three more cases to the literature on this subject. ICAc/o is caused by a spread of the inflammatory process to the CS, followed by ICA compression and probable thickening of the arterial wall. The recurrence of hypophysitis, optomotor nerve palsy, and the parasellar T2 dark sign should alert the treating physicians to the possible involvement of the ICA. Special attention to the carotid arteries is warranted because ICAo is a severe sequela of hypophysitis associated with a considerable risk of adverse neurological outcomes.

## Declaration of interest

The authors declare that there is no conflict of interest that could be perceived as prejudicing the impartiality of the work reported.

## Funding

This work did not receive any specific grant from any funding agency in the public, commercial, or not-for-profit sector.

## Ethics approval

The study was approved by the ethics committee of the University of Tuebingen (number: 230/2023BO2).
